# Exploring the knowledge and skills for effective family caregiving in elderly home care: a qualitative study

**DOI:** 10.1186/s12877-024-04924-3

**Published:** 2024-04-15

**Authors:** Gebrezabher Niguse Hailu, Muntaha Abdelkader, Feven Asfaw, Hailemariam Atsbeha Meles

**Affiliations:** 1 School of Nursing, Mekelle University, Mekelle, Ethiopia; 2 School of Public Health, Department of Health Informatics, Mekelle University, Mekelle, Ethiopia

**Keywords:** Family caregiving, Elderly home care, Knowledge, Skills, Qualitative study

## Abstract

**Background:**

Family caregivers play a crucial role in providing physical, emotional, and social support to the elderly, allowing them to maintain their independence and stay in their preferred living environment. However, family caregivers face numerous challenges and require specific knowledge and skills to provide effective care. Therefore, understanding the knowledge and skills required for effective family caregiving in elderly home care is vital to support both the caregivers and the elderly recipients.

**Methods:**

The research was carried out in Mekelle City, Ethiopia, utilizing the phenomenology study design and purposive sampling technique. A total of twenty-two in-depth interviews were conducted. Individuals with experience in providing care for elderly people in their homes were targeted. Data was gathered through the use of an open-ended guide, transcribed word-for-word, inputted into ATLAS.ti8 software, and translated. Codes and themes were then extracted from the transcribed data, and a thematic analysis was performed. To minimize personal biases, the collected data were coded independently by the data collection assistants and the PI. The analysis was carried out by authors who were not involved in the data collection process. The interviews were conducted in a quiet place.

**Results:**

A total of 22 in-depth interviews were conducted as part of this research. The results indicated that although the participants had knowledge about common health problems experienced by older people, they were uninformed about how to manage these conditions at home and were unaware of specialized healthcare resources for the elderly. Furthermore, they had limited knowledge about suitable exercise routines, strategies to prevent falls, and home healthcare practices for older individuals. On the other hand, they exhibited a solid comprehension and awareness of abusive behaviors specifically directed at older adults.

**Conclusion:**

The results emphasized the importance of enhancing education and training for family caregivers in handling elderly health issues, raising awareness about specialized healthcare services catered to the elderly, improving understanding of activities of daily living (ADLs) and fall prevention, and offering inclusive training in healthcare tasks related to elder care.

**Recommendation:**

Participants should receive comprehensive education and training programs to enhance their knowledge and skills in managing these conditions. Efforts should also be made to raise awareness about the availability of geriatric hospitals or specialized nurses for the elderly. Participants need to be educated about suitable exercise routines for the elderly and fall prevention strategies. Healthcare skills training is also necessary for participants, focusing on activities such as wound dressing, vital sign monitoring, and establishing a specific schedule for changing positions.

## Introduction

Healthy aging is a constant process that improves the person’s health and quality of life [1].

According to a UN estimate on the world’s population outlook, by 2050, more than 63% of the world’s population—or approximately 22% of all people—will be 60 years of age or older and more than 63% will reside in Africa [2].

Caregiving becomes increasingly complicated and difficult as people age because of chronic illnesses and ongoing loss of mental and physical independence [3].Furthermore, research indicates that more than 70% of persons 60 and older view old age as a nightmare, a time of uncertainty about the future, and a time when they must rely on others for nearly everything [4].

The family-centered care paradigm was famously created in pediatrics and has now been applied to various fields, such as cancer, HIV, palliative care, and hospice care [5]. It is currently being modified for the care of vulnerable older individuals. Through information exchange, involvement in decision-making, and cooperation in the formulation of policies and programs, patient and family knowledge, values, beliefs, and cultural backgrounds are incorporated into the planning and delivery of treatment [6].

The older adult population suffers from poverty and social isolation especially in developing country like Ethiopia since many of them are retired and have scant or no retirement plans or benefits. For example, a study on the elderly conducted in Cameron revealed that older adults are the most vulnerable and disadvantaged people in society and are reliant on family members for their fundamental requirements [7].

Elderly family members who are unable to take care of themselves are supported by family caregivers who offer them physical, emotional, psychological, and occasionally financial support [8]. The majorities of them, however, perform this duty with little to no understanding of care, assistance, or support [9] Family caregivers who lack sufficient understanding may unintentionally injure their loved ones and sometimes even themselves [10, 11].

In sub-Saharan Africa, it is typical for the younger spouse, (often the wife), children, or grandkids to be responsible for caring for their elderly family members [12].These family carers are required to help their elderly family members with everyday tasks and guard against elder abuse and falls [13–15].More broadly, they aid older family members who are unable to care for themselves by providing physical, emotional, psychological, and occasionally financial support [8].

Ethiopian traditional beliefs presuppose that younger family members will care for the elderly. Families, including children and relatives, are expected to care for the elderly, thus helping them is seen as a blessing and a good opportunity for kids. However, the majority of the family care givers in sub-Saharan Africa, including Ethiopia carry out this function with little to no knowledge of care, aid, or support [8, 9]. Thus, exploring family care giver’s knowledge and skills for effective family caregiving in elderly home care using qualitative study to gather rich and detailed information that goes beyond numerical data is paramount important to take further action about caring of older adults at home.

## Methods

### Study area and period

The research took place in Mekelle, which is a city located in the Tigray region of northern Ethiopia. The study was conducted between December 2022 and October 2023. Mekelle is both the capital and largest city in the region, with a population of about 500,000 residents. The city is situated at an altitude of 2,084 m (6,837 feet) above sea level and is approximately 783 km (487 miles) away from the Ethiopian capital of Addis Ababa.

### Study design

Phenomenological Study design was used to explore the personal experiences, thoughts, feelings, and perceptions of family caregivers in the context of providing care for elderly individuals at home.

### Source and study populations

#### Source population

All individuals who are over the age of 18 and living in Mekelle city and responsible for taking care of their elderly relatives in their homes.

#### Study population

All selected family care givers who were responsible for providing care for their elderly loved ones within their own homes.

#### Sample size determination

The sample size was determined by the level of saturation. Twenty two study participants were used for in-depth interview.

### Sampling technique and procedure

We used a purposive sampling technique, which is non-probability, to select participants for our study. We specifically targeted individuals with experience in providing care for elderly people in their homes. To find these participants, we reached out to healthcare professionals like doctors, nurses, and social workers who regularly interact with elderly patients. Community events related to elderly care and family caregiving also allowed us to connect with potential participants. Additionally, we made use of online platforms, such as social media groups focused on family caregiving or elderly care, in order to reach a larger audience.

### Data collection method

We developed and translated an open-ended English guide into the local language, Tigrigna, with the assistance of experts. Two experienced research assistants helped with the data collection process as data collection assistants. One assistant used a tape recorder to record interviews, while the other took notes. The PI moderated the data collection process. In-depth interviews were conducted to gather thorough information on the experiences, perspectives, and insights of family caregivers who provide care for elderly individuals in their homes.

### Data quality assurance

Before starting the actual work, open-ended guiding questions were prepared and discussed with experts. Data collection assistants were trained to take notes and record using a tape recorder for one day. The use of an open-ended guiding question helped prevent dominance from participants. After each day of data collection, a debriefing session was conducted by the data collection assistants and the principal investigator (PI). The recorded data were read, re-read, and transcribed by both the researchers and data collection assistants separately to ensure data reliability. To minimize personal biases, the collected data were coded independently by the data collection assistants and the PI. The analysis was carried out by authors who were not involved in the data collection process. The interviews were conducted in a quiet place.

### Data analysis and presentation

The information was gathered using a tape recorder and notes. It was later transcribed, entered into Atlas-ti8, and translated. Codes were generated and organized, eventually leading to the formation of five themes. The thematic analysis approach was employed, and the outcome was presented in both written form and as a table.

## Results

A total of twenty two individuals were participated in the study (Table 1). The results of the study were summarized in to five thematic areas: Awareness of participants on common elderly health conditions and their home based management care, Awareness of study participants on availability of elderly care resources, Awareness on practice of ADLs and fall prevention, awareness towards practices related to simple and highly skilled healthcare activities, and awareness on abuse related practices.

### Awareness of participants towards common health conditions and their home based management care

Concerning the awareness of participants regarding common health conditions in elders, participants showed a good understanding. They mentioned nearly all of the common health conditions that affect elders. However, most participants lacked knowledge about how to manage these conditions properly. Caregivers, based on their opinions, have a widespread awareness of the common health conditions that affect elderly individuals. Conditions such as joint pain, forgetfulness and memory loss, visual impairment, hearing loss, and age-related muscle weakness were among those mentioned. However, what is particularly notable is the lack of knowledge regarding the management of these conditions. The participants expressed uncertainty about selecting appropriate exercises for joint pain, effectively managing forgetfulness and memory loss, providing assistance beyond clear communication for hearing loss, and measuring blood pressure.

*“…….Joint pain is a common health condition among the elderly. My mother often complains about her knees and hip joints hurting, particularly during long periods of walking or standing. While I provide her with exercises, I am unsure about the most appropriate type of exercise to alleviate her pain. I know also some other common elderly health conditions like diabetes, high blood pressure and eye problems…….P*_*1*_*”*.

*“I have observed instances of forgetfulness and memory loss not only in my father but also in several other elderly individuals. It is quite common for him to misplace his belongings frequently or repeatedly ask the same question……… This issue poses a significant challenge for me as I am uncertain about how to effectively manage and assist him with this problem on my own. Additional support and guidance are desperately needed in order to address this situation adequately…P*_7_*”.*

*“My father is experiencing respiratory conditions, and I am also aware that high blood pressure is common among older individuals……. I have noticed several instances where it remains undetected until it causes significant health problems……….Unfortunately, we do not have a blood pressure measuring device at home and I have no any clue how to measure it, so I am unable to check my father’s blood pressure. However, when I take him to the hospital for his other health concerns, they do measure his blood pressure during the check-up….P*_*10*_*”*.

One participant demonstrated a good understanding and proactive approach towards managing his grandmother’s visual impairment caused by age-related eye problems. But nevertheless, the participant lacked knowledge and understanding on management of other common health conditions experienced by older adults such as joint pain, neck and back pain, memory loss, and chronic diseases.

*“…………….My grandmother suffers from visual impairment caused by age-related eye problems. She experiences difficulty in reading or recognizing faces from a distance. To effectively manage this condition, I ensure her eyeglasses are clean and take her for regular eye check-ups. I know also many other cases which are common in older adults although, I have no clue about their management. For example: joint pain, neck and back pain, memory loss, and chronic diseases….P*_*6*_*”*.

Another one participant has also demonstrated awareness and proactive measures in managing his father’s health condition related to age-related muscle weakness. By installing handrails and providing a walker, he has significantly improved his father’s mobility and independent functioning.

*“My father has difficulties walking due to age-related muscle weakness. He often requires assistance with activities like climbing stairs or getting in and out of bed. I have installed handrails around the house and provided him with a walker, significantly improving his mobility…P*_*15*_.*”*

### Awareness of study participants on availability of Elderly Care resources

The participants have expressed a lack of awareness regarding the availability of geriatric hospitals or specialized nurses for the elderly. They acknowledged their limited knowledge and stated that they had not come across any specific resources or services catering to elderly care. *“……………As far as my knowledge goes, I don’t think there are geriatric hospitals. I haven’t come across any information or services specifically focused on elderly care. I know there are general hospitals and nurses, but I have no idea if there are specific geriatric hospitals or specialized nurses for the elderly….P*_*2*_*”*.

However, they were aware of the presence of non-governmental organizations and social care services that offer assistance and support for the elderly population.

*“Yes, I was aware of the existence of non-governmental organizations and social care services that offer assistance and care for the elderly. In fact, I have personally utilized some of these services for my aging parents………. These organizations provide various forms of support, such as in-home care, transportation assistance, and respite services. They have been a valuable resource for my family, helping us ensure that our elderly loved ones receive the necessary care and support they need…… P*_*4*_*”*.

*“………….Certainly, I have knowledge about the presence of non-governmental organizations and social care services that offer assistance and care for the elderly. When my grandmother required more support than our family could provide on our own, we explored these services and found several reputable organizations that support elders. They offer services such as personal care, meal preparation, and even companionship for the elderly. The availability of these organizations has been a great relief for families like ours, as they provide professional caregivers who contribute to the overall well-being and quality of life of the elderly population……P*_*8*_*”*.

### Awareness on practices of ADLs and fall prevention

Study participants were actively involved in assisting their elder family members with certain activities of daily living (ADLs) such as cooking meals, washing clothes, and bathing.

*“……….As a family caregiver, I take care of my aging loved one by cooking meals, washing their clothes, and ensuring they receive proper assistance with bathing….P*_*16*_.*”*

*“I have taken on the responsibility of cooking meals, washing my elderly family member’s clothes, and helping them with bathing to ensure their comfort and well-being in their home….P*_*1*_.*”*

But nevertheless, they lacked knowledge about suitable exercise routines for the elderly and, therefore, do not provide any assistance in that aspect.

*“……I didn’t know that there were limits to the elderly’s exercise capacity. I just assumed they would let me know if something was too much for them. I never actively engaged them in exercise or encouraged any physical activities….P*_*17*_.*”*

*“Exercise routines?…. I guess I never thought much about it. I mean, they’re old, so I didn’t think they need much physical activity. I haven’t made any specific measures to ensure their safety or meet their needs in terms of exercise….P*_*22*_.*”*

One has expressed with limited awareness of home arrangements with the elderly consideration.

*“….To be honest, I never really thought about the safety aspects of the home in relation to the elderly. I didn’t make any specific changes or modifications to prevent accidents or accommodate their needs…P*_*13*_*”*.

### Awareness towards practices related to simple and highly skilled healthcare activities

Participants in the study fulfilled their duty of looking after their elderly family member by complying with the doctor’s instructions and ensuring that the recommended medications were taken as instructed. Their dedication involved following the prescribed dosage and timing to ensure their family member derived the utmost advantage from the medication. Additionally, the participants showed their support by accompanying the elderly family member to medical appointments.

*“I always make sure to give the prescribed medications to my elderly mother member as per the doctor’s instructions………. I try my best to follow the dosage and timing mentioned on the prescription…P*_*18*_*”*.

*“……………Taking my elderly family member for doctor’s appointments is an important responsibility for me. I make sure to accompany them and ask relevant questions to the doctor regarding their health condition and medication…….P*_*10*_ *”.*

However, they lacked knowledge and skills in home based healthcare activities such as wound dressing, monitoring vital signs, and establishing a specific schedule for changing positions. While they strived to meet their family members’ requests for position changes and the changing of soiled linens, they were not necessarily adhering to a specific time interval.

No, I am not aware of any specific healthcare activities related to wound dressing or changing positions frequently to prevent becoming bedridden. I only change the position when my elderly family member asks for it and change the soiled linens as soon as they get soiled.P_7_.

*“I don’t have the knowledge or skills to monitor vital signs or perform wound dressing at home. I do change the positions of my elderly family member when they request it, but I am not aware of the specific time intervals when to change….P*_*20*_*”*.

### Awareness on abuse related practices

The care givers had a strong awareness and understanding of abuse-related practices towards elders. They condemn any form of physical abuse, such as beating, and emphasize the importance of communication and peaceful resolution of conflicts. They also recognize the significance of providing proper nourishment and addressing dietary needs, highlighting the harm of neglecting the elderly’s access to healthy meals. Respectful and calm communication is advocated to avoid any emotional or mental harm to elders. Additionally, they stress the responsibility of providing attention and care, rejecting the notion of ignoring elders.

*“I believe that abuse towards our elders is completely unacceptable……… I have never resorted to beating my elderly parents as a means of control or discipline. Instead, I try to communicate with them calmly and find peaceful solutions to any issues that may arise….P*_*3*_.*”*

*“…………Refusing food to our elders is a form of neglect and abuse. I strongly believe in providing nourishment and taking care of their dietary needs. It is vital to ensure that they have access to healthy and balanced meals to maintain their well-being….P*_*21*_*”*.

*“Yelling at our elders is not only disrespectful but also harmful to their mental and emotional health. I always try to maintain a calm and patient demeanor when communicating with my elders, even in challenging situations….P*_*9*_*”*.

*“I believe it is our responsibility to provide proper attention and care to our elders. Ignoring them is simply not an option…………. I dedicate time every day to spend quality moments with them, listen to their stories, and address their concerns….P*_*11*_.*”*

## Discussion

The findings of this qualitative study highlight several important gaps in knowledge and skills among family caregivers in elderly home care. The participants demonstrated a good understanding of common health conditions affecting elders, indicating a basic foundation of knowledge in this area. However, they lacked knowledge about proper management of these conditions, suggesting a need for education and training to improve their caregiving abilities. This finding aligns with previous research, which has also identified knowledge gaps among family caregivers in areas such as medication management and symptom control [16].

One notable gap identified in this study is the lack of awareness regarding the availability of geriatric hospitals or specialized nurses for the elderly. This suggests that participants may be unaware of the resources and support services available to them, which could impact their ability to provide optimal care for their elderly relatives. Improving awareness of these resources is therefore crucial to ensure that family caregivers are accessing the necessary support to fulfill their caregiving responsibilities effectively. Previous studies have also highlighted the importance of providing information and education to family caregivers about available resources and services [17].

Another significant gap identified in this study is the lack of awareness and knowledge about suitable exercise routines for the elderly activities on daily living (ADLs) and fall prevention. ADLs are essential for maintaining independence and quality of life among elderly individuals, and family caregivers have a crucial role in assisting with these activities. Similarly, fall prevention is vital to prevent injuries and maintain the well-being of elderly individuals. Addressing this knowledge gap is essential to ensure that family caregivers are equipped with the necessary skills to promote independence and prevent accidents. Previous studies have emphasized the importance of caregiver education and training in ADLs and fall prevention [18].

Furthermore, participants in this study exhibited insufficient knowledge and skills in healthcare activities such as wound dressing, monitoring vital signs, and establishing a specific schedule for changing positions. These activities are critical for maintaining the health and well-being of elderly individuals, particularly those with chronic illnesses or physical limitations. Providing education and training on these healthcare activities is crucial to enhance the caregiving skills of family caregivers and ensure the provision of adequate medical care at home.

Despite these knowledge and skills gaps, it is encouraging to note that most participants demonstrated a strong awareness and understanding of abuse-related practices towards older adults. This highlights the importance of recognizing and addressing elder abuse, as family caregivers are in a unique position to detect and prevent such abuse. However, further research is needed to explore the specific actions and strategies that family caregivers can employ to effectively address elder abuse.

## Conclusion

The results indicate that while participants showed a good understanding of common health conditions affecting elders, they lacked knowledge about managing these conditions properly. Participants also expressed a lack of awareness regarding the availability of geriatric hospitals or specialized nurses for the elderly. There was also a lack of awareness and knowledge about suitable exercise routines for the elderly on activities of daily living (ADLs) and fall prevention. Furthermore, participants lacked knowledge and skills in healthcare activities such as wound dressing, monitoring vital signs, and establishing a specific schedule for changing positions. However, most participants had a strong awareness and understanding of abuse-related practices towards elders. In conclusion, the study reveals that participants possess a solid understanding of common health conditions affecting the elderly but lack knowledge about proper management of these conditions. Furthermore, participants expressed a lack of awareness regarding the availability of geriatric hospitals or specialized nurses for the elderly. Moreover, there was a notable lack of awareness and knowledge regarding practices related to activities of daily living (ADLs) and fall prevention. Additionally, participants exhibited insufficient knowledge and skills in healthcare activities such as wound dressing, monitoring vital signs, and establishing a specific schedule for changing positions. Despite these gaps, most participants demonstrated a strong awareness and understanding of abuse-related practices towards older adults.

### Recommendation

Participants should receive comprehensive education and training programs to enhance their knowledge and skills in managing these conditions. This includes learning proper management techniques, understanding available healthcare resources, and being aware of specialized care options for older adults. Efforts should also be made to raise awareness about the availability of geriatric hospitals or specialized nurses for the elderly. Participants need to be educated about suitable exercise routines for the elderly and fall prevention strategies. Healthcare skills training is also necessary for participants, focusing on activities such as wound dressing, vital sign monitoring, and establishing a specific schedule for changing positions.

**Table 1:** Socio demographic characteristics of study participants n(22)

Variables	Category	Frequency
Age	26–44	8
	45–59	14
Gender	Male	8
	female	14
Marital status	Married	19
	Single	2
	Divorced	1
Kebelle	Ayder	7
	Adishumdhun	4
	05	5
	Adihaki	1
	Adihawsi	2
	17	2
	11	1

NB: Kebelle is a place where the study participants reside in or came from.

## Data Availability

The datasets used and/or analysed during the current study available from the corresponding author on reasonable request. •.

## Abbreviations

ADLs: Activity of Daily living
HIV: Human immune virus
IRB: Institutional review board
PI: Principal investigator
UN: United Nation
